# Characterization of the complete mitochondrial genome of *Inara alboguttata* (Hemiptera: Reduviidae)

**DOI:** 10.1080/23802359.2018.1511857

**Published:** 2018-10-08

**Authors:** Siyu Gong, Fan Song, Hu Li

**Affiliations:** Department of Entomology and MOA Key Lab of Pest Monitoring and Green Management, College of Plant Protection, China Agricultural University, Beijing, China

**Keywords:** Mitochondrial genome, reduviidae, *Inara alboguttata*

## Abstract

In this study, the complete mitochondrial genome (mitogenome) of the assassin bug *Inara alboguttata*, is determined using next-generation sequencing. The mitogenome is a typical circular DNA molecule of 15,436 bp long with a high AT bias (72.3%), containing 13 protein-coding genes, two rRNA genes, 22 tRNA genes and a control region. Protein-coding genes all initiate with ATN codons and most of them terminate with TAA or TAG codons, whereas *COI*, *ND3*, and *ND5* use a single T residue. The *lrRNA* and *srRNA* genes are 1247 bp and 766 bp in length, respectively. All tRNA genes have the cloverleaf secondary structure except for the *tRNA^Ser(AGN)^.* The control region is 860 bp long with an A + T content of 68.2%. Phylogenetic result supports the sister relationship between *I. alboguttata* and *Acanthaspis ruficeps*.

*Inara* Stål is a small genus within the subfamily Reduviinae (Hemiptera: Reduviidae) including six known species, and all of them are distributed in the Oriental regions (Maldonado-Capriles 1990). In this study, we characterized the complete mitochondrial genome (mitogenome) sequence of *Inara alboguttata,* the first representative of the genus *Inara* and performed an analysis of the phylogenetic relationships among Reduviidae with the available mitogenomic sequences.

Total genomic DNA was extracted from the thorax of adult collected from Nonggang National Nature Reserve in Guangxi province, China. Voucher specimen (No. VCim-00107) was deposited at the Entomological Museum of China Agricultural University (CAU). The complete mitogenome was obtained by next-generation sequencing method with Illumina Hiseq 2500 and the sequence was deposited in GenBank under the accession number KY069959.

The whole mitogenome is a circular DNA molecule of 15,436 bp long, including 37 genes (13 protein-coding genes, 22 tRNA genes and two rRNA genes) and a control region. The gene arrangement is largely the same as the putative ancestral arrangement of insects and most assassin bugs (Li et al. 2011, 2015; Gao et al. 2013; Zhao et al. 2015; Zhang et al. 2016). The overall nucleotide composition of the whole mitogenome is significantly biased toward A + T (72.3%) with positive AT-skew (0.14) and negative GC-skew (−0.16). All protein-coding genes initiate with ATN as the start codon (three with ATT, five with ATA and five with ATG). Conventional stop codons (TAA or TAG) have been assigned to majority of the protein-coding genes. *COI*, *ND3*, and *ND5*, however, terminate with a single T residue.

This mitogenome has the complete set of 22 tRNA genes, ranging from 61 to 70 bp. All of the tRNA genes have a typically cloverleaf structure except for the dihydrouridine (DHU) arm of *tRNA^Ser(AGN)^* forms a simple loop. The *lrRNA* is 1247 bp long with an A + T content of 74.5%, and the *srRNA* is 766 bp long with an A + T content of 72.2%. The control region located between *srRNA* and *tRNA^Ile^* is 860 bp long with an A + T content of 68.2%. Tandem repeats of two 27-nt units are found in the control region.

Maximum-likelihood (ML) tree was constructed based on sequences of 13 protein-coding genes and two rRNA genes from 16 Reduviidae species by using RAxML-HPC2 (Stamatakis 2014) under the GTR + I + G model (Figure 1). The sister relationship between *I. alboguttata* and *Acanthaspis ruficeps* is highly supported. However, *Reduvius tenebrosus* is the sister group to *Valentia hoffmanni* (Salyavatinae), therefore, the monophyly of Reduviinae is not recovered, which is similar to the results of previous studies (Weirauch and Munro 2009; Hwang and Weirauch 2012; Jiang et al. 2016).

**Figure 1.**
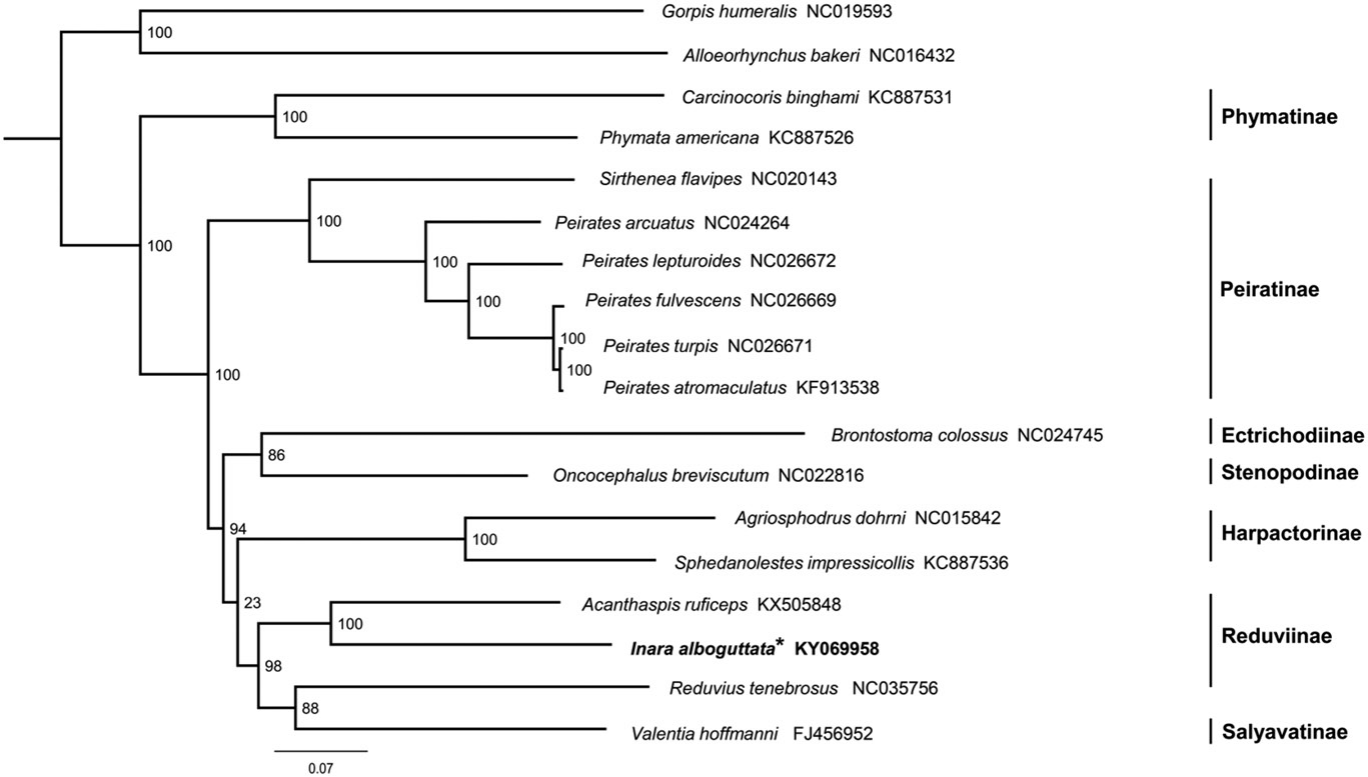
Phylogenetic tree inferred from ML analysis of the nucleotide of the 13 protein-coding genes and two rRNA genes (12,697 bp). The nodal values indicate the bootstrap percentages obtained with 1000 replicates. GenBank accession numbers for the sequences are indicated next to species name. Mitogenome newly sequenced in the present study is highlighted by the asterisk.
